# Supplementary material for: Concerted localization-resets precede YAP-dependent transcription
Source: Nat Commun. 2020 Sep 11;11:4581. doi: 10.1038/s41467-020-18368-x (PMC7486942; doi:10.1038/s41467-020-18368-x)
Supplement: Supplementary file 1 — Supplementary Information [file 41467_2020_18368_MOESM1_ESM.pdf]

Supplementary Figures

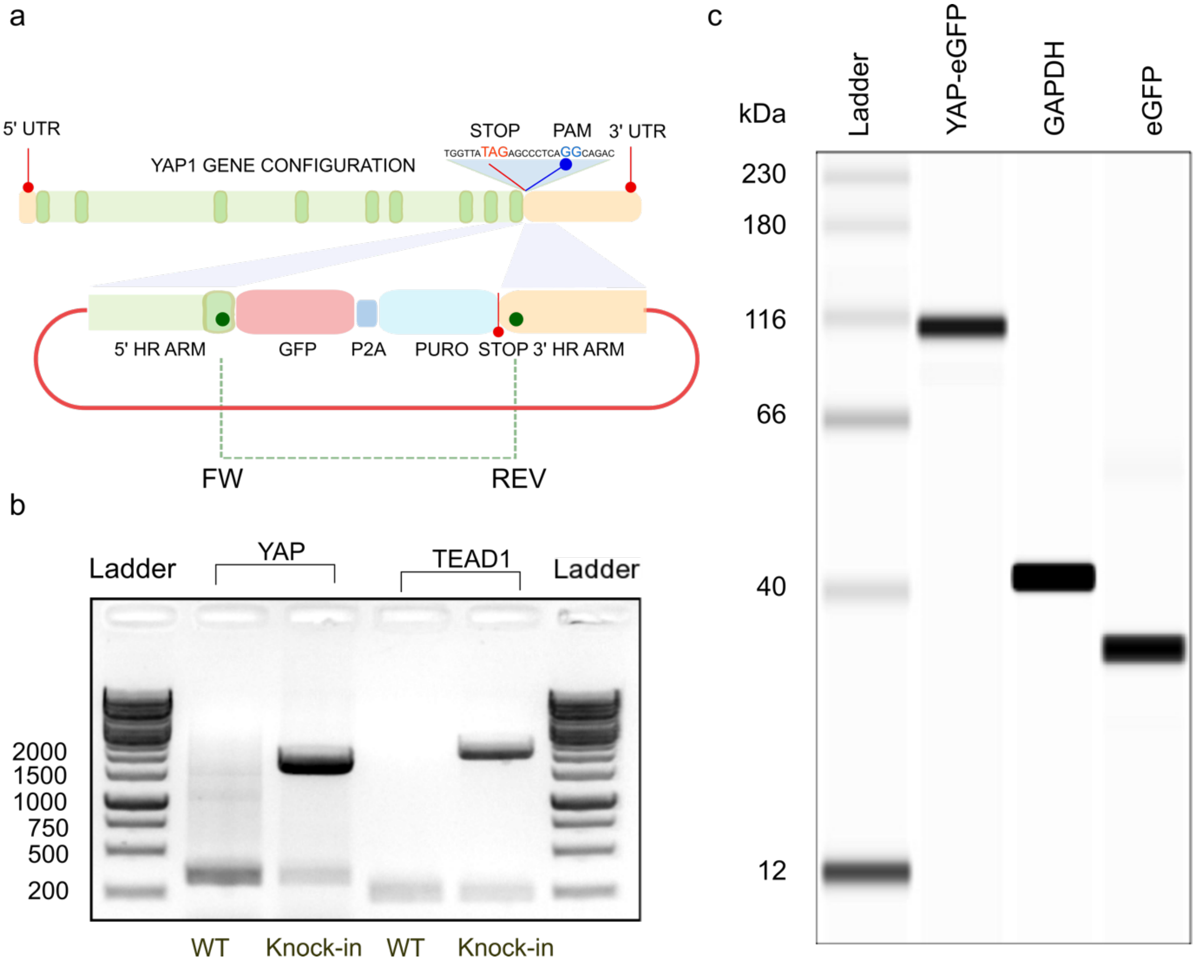

Supplementary Fig. 1: Validation of Fluorescent Protein knockin cell lines. a. Top: Cartoon of CRISPR-Cas9 based insertion of eGFP-P2A-puromycin cassette at 3' end of the YAP gene as shown in **main text Figure 1a**. Two sites in the 5' and 3' HR arms are marked to show locations of the FW and Rev primers used for PCR based validation of knock-in. Similar primer design was used to validate mCherry-P2A-Hygromycin cassette knockin at the 3' end of the TEAD1 gene. **b.** Agarose gel electrophoresis of amplicons produced by PCR using YAP (lanes 2, 3) and TEAD1 (lanes 4, 5) primers sets against total genomic DNA extracted from WT (wildtype) and KI (knock-in) MCF10A cells. **c.** Capillary western blot using anti eGFP (lane 2) and anti GAPDH (lane 3) antibodies against MCF10A$^{YAP\text{-}GFP\text{-}KI}$ whole cell lysate, and anti eGFP antibody against recombinant eGFP (lane 4). Observed vs expected (theoretical) sizes: Yap-eGFP (110/100), GAPDH (43/36), eGFP (35/29).

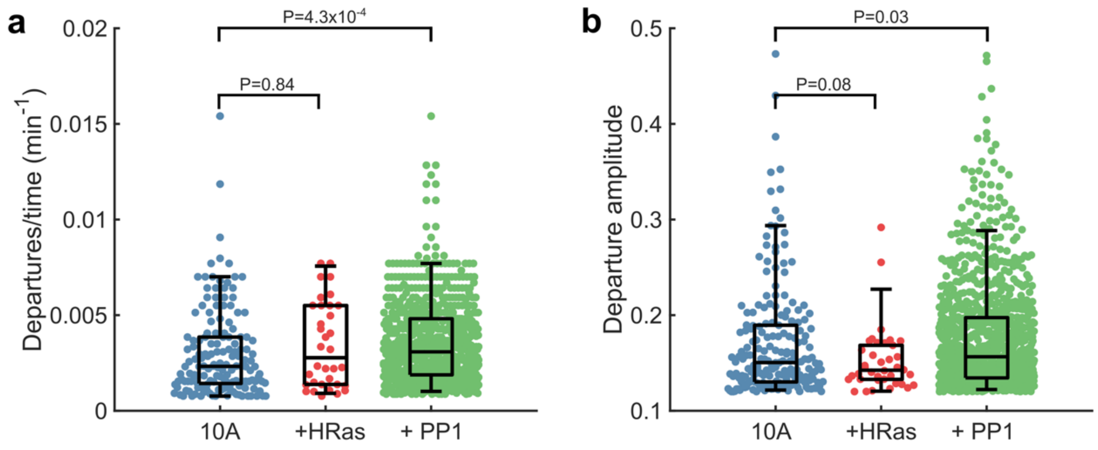

Supplementary Fig. 2. YAP N/C pulse density and amplitude analysis under different conditions (10A, 10A+HRas, 10A+PP1). Box and whisker lines: black-center line is median value, box-bounds are 25th and 75th percentile, and whiskers are 0.05 and 0.95 percentiles. Data compiled from two independent 24-hr experiments for each condition. P-values are from two-sided Mann-Whitney U-test to test whether fluctuation frequency or amplitude is changed from MCF10A. (**a**) *Frequency*: N_{10A}=150, N_{HRas}=34, N_{PP1}=693 tracks. 10A vs +HRas (P=0.84, Ranksum=13596, Z-val=-0.99), 10A vs +PP1 (P=4.33x10^{-4}, Ranksum=54295, Z-val=-3.33). (**b**) *Amplitude*: N_{10A}=192, N_{HRas}=38, N_{PP1}=970. 10A vs +HRas (P=0.08, Ranksum=22683, Z-val=1.35), 10A vs +PP1 (P=0.03, Ranksum=103896, Z-val=-1.8245).

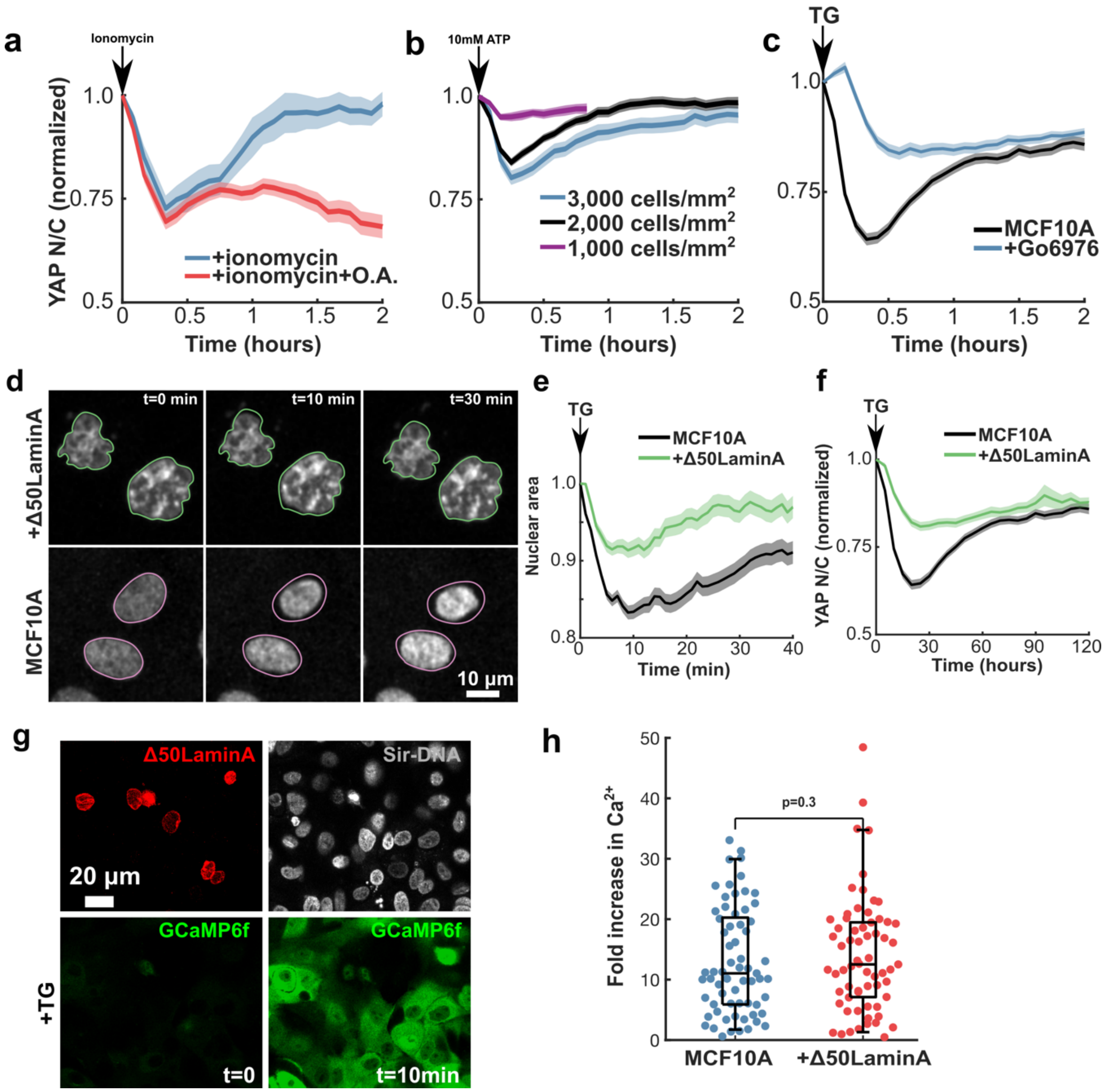

Supplementary Fig. 3: Mechanism of YAP spatial reset. (**a-c**) Mean YAP N/C for cells tracked over time under various conditions (All data are normalized to initial time point). Solid line is mean and shaded area is S.E.M. (**a**) Treatment with ionomycin (N=47 tracks) or ionomycin and Okadaic acid (N=104 tracks). (**b**) Treatment with 10mM ATP at cell densities of 1,000 cells/mm^2 (N=80 tracks), 2,000 cells/mm^2 (N=162 tracks), and 3,000 cells/mm^2 (N=186 tracks). (**c**) Go6976 pre-treatment + TG (N=104 tracks). Data in **a**-**c** are aggregated from two independent experiments for each condition. (**d**) Representative images of the nuclear compression following release of calcium with TG in MCF10A (left column) and MCF10A co-expressing Δ50LaminA (right column). (**e**) Normalized projected nuclear area for MCF10A cells (N=166 tracks), and MCF10A cells co-expressing Δ50LaminA (N=87 tracks) upon treatment with TG. Data in **b** and **c** are aggregated from two independent experiments. Solid line is mean and shaded area is S.E.M. (**f**) Mean YAP N/C (normalized to initial time point) for MCF10A cells (N=137 tracks) and MCF10A cells co-expressing Δ50LaminA (N=106 tracks) upon treatment with TG. Solid line is mean and shaded area is S.E.M. (**g**) Representative images of Thapsigargin-induced Ca^{2+} release. *Top:* Transient transfection of GCaMP6f cell line with Δ50LaminA-mCherry and cell nuclei stained with Sir-DNA. *Bottom*: GCaMP6f reporter with TG-treatment at t=0 and t=10 minutes. (**h**) Fold-increase in Ca^{2+} as measured by GCaMP6f signal at t=10 minutes normalized to mean signal at t=0. $N_{control}$=65, N_{Lamin}=65 cells. Box and whisker lines: black-center line is median value, box-bounds are 25th and 75th percentile , and

whiskers are 0.05 and 0.95 percentiles. P=0.3 from two-sided Mann-Whitney U-test (zval: -0.5262, ranksum: 4144). Repeated three times with similar results.

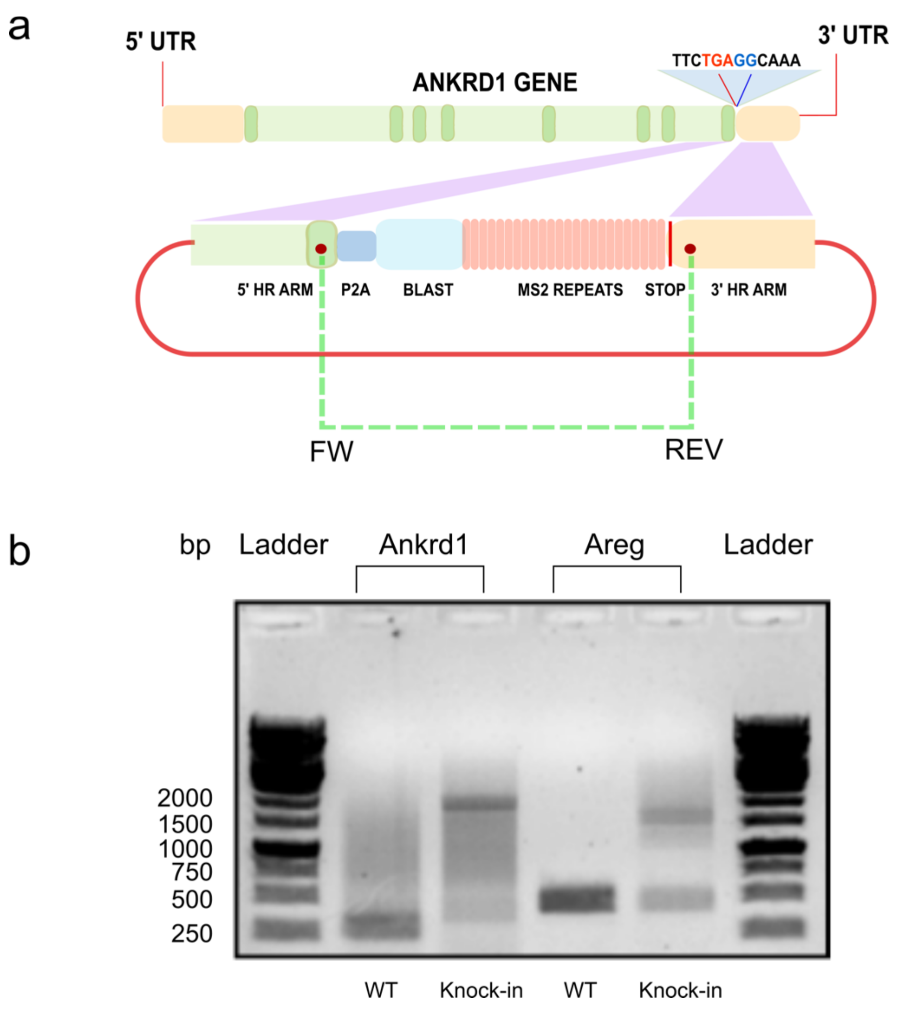

Supplementary Fig 4: Validation of MS2 (transcriptional reporter cassette)-knockin cell lines. a. Top: Cartoon of CRISPR-Cas9 based strategy for knocking in an MS2 transcription reporter cassette at the end of the coding sequence of ANKRD1 gene as shown in main text Figure 5a. Two sites in the 5' and 3' HR arms are marked to show locations of the FW and Rev primers used for PCR based validation of knock-in. Similar primer design was used to validate MS2 cassette knockin at the the end of the coding sequence of AREG gene.

b. Agarose gel electrophoresis of amplicons produced by PCR using Ankrd1 (lanes 2, 3) and AREG (lanes 4, 5) primers sets against total genomic DNA extracted from WT (wild type) and KI (knock-in) MCF10A cells.

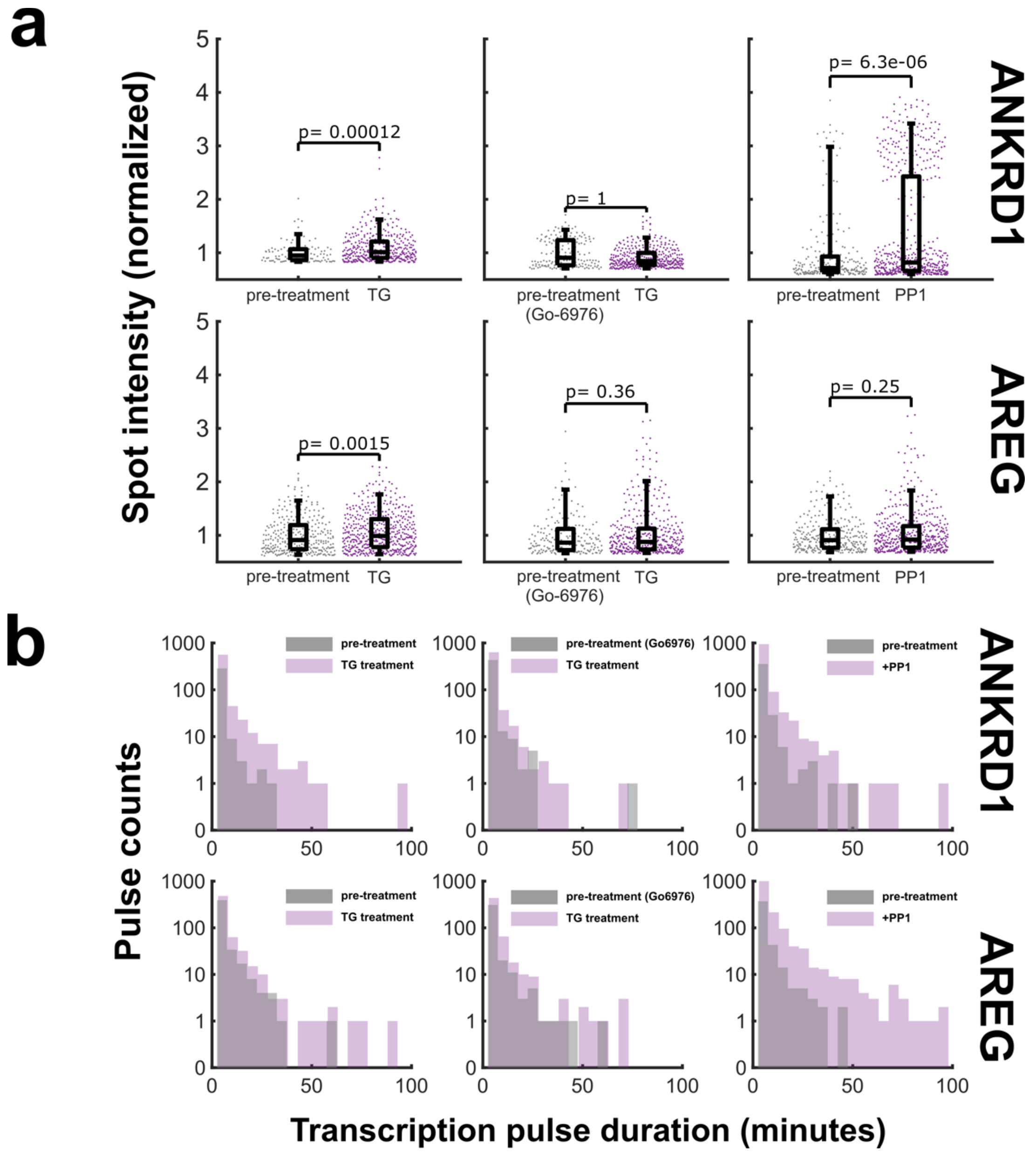

Supplementary Fig. 5: Intensity and duration analysis of AREG and ANKRD1 gene transcription pulses under various conditions. (**a**) Transcription spot intensity comparison between pre-treatment period (‘control’) and post-treatment period (“TG” or “PP1”). Spot intensity is the integrated fluorescence intensity pixel counts of each detected spot after subtracting local background signal. For each individual experiment, the transcription amplitude was normalized to the pre-treatment period such that the mean intensity is 1. The one-sided Mann-Whitney U-test P-value is reported for each pre-treatment: post-treatment comparison. *ANKRD1:* TG (P=1.2x10-4, Ranksum=20359, Z-val=-3.66, $N_{control}$=105, N_{TG}=368), +Go6976 (P=1.0, Ranksum=51121, Z-val=3.2, $N_{control}$=168, N_{TG}=375), +PP1 (P=6.3×10^{-6}, Ranksum=74535, Z-val=-4.4, $N_{control}$=229, N_{PP1}=526). *AREG*: TG (P=0.0015, Ranksum=114063, Z-val=-2.95, $N_{control}$=309, N_{TG}=489), +Go6976 (P=0.36, Ranksum=56696, Z-val=-0.35, $N_{control}$=191, N_{TG}=409), +PP1 (P=0.25, Ranksum=114648, Z-val=-0.67, $N_{control}$=297, N_{PP1}=488). The distribution was generated by compiling data from all experiments from each condition. Box and whisker lines: black-center line is median value, box-bounds are 25th and 75th percentile , and whiskers are 0.05 and 0.95 percentiles. (**b**) The distribution of pulse-durations are shown for each pre-treatment: post-treatment comparison, as in **a**. Pulse duration is defined as the time over which a nascent transcription spot was detectable and tracked.

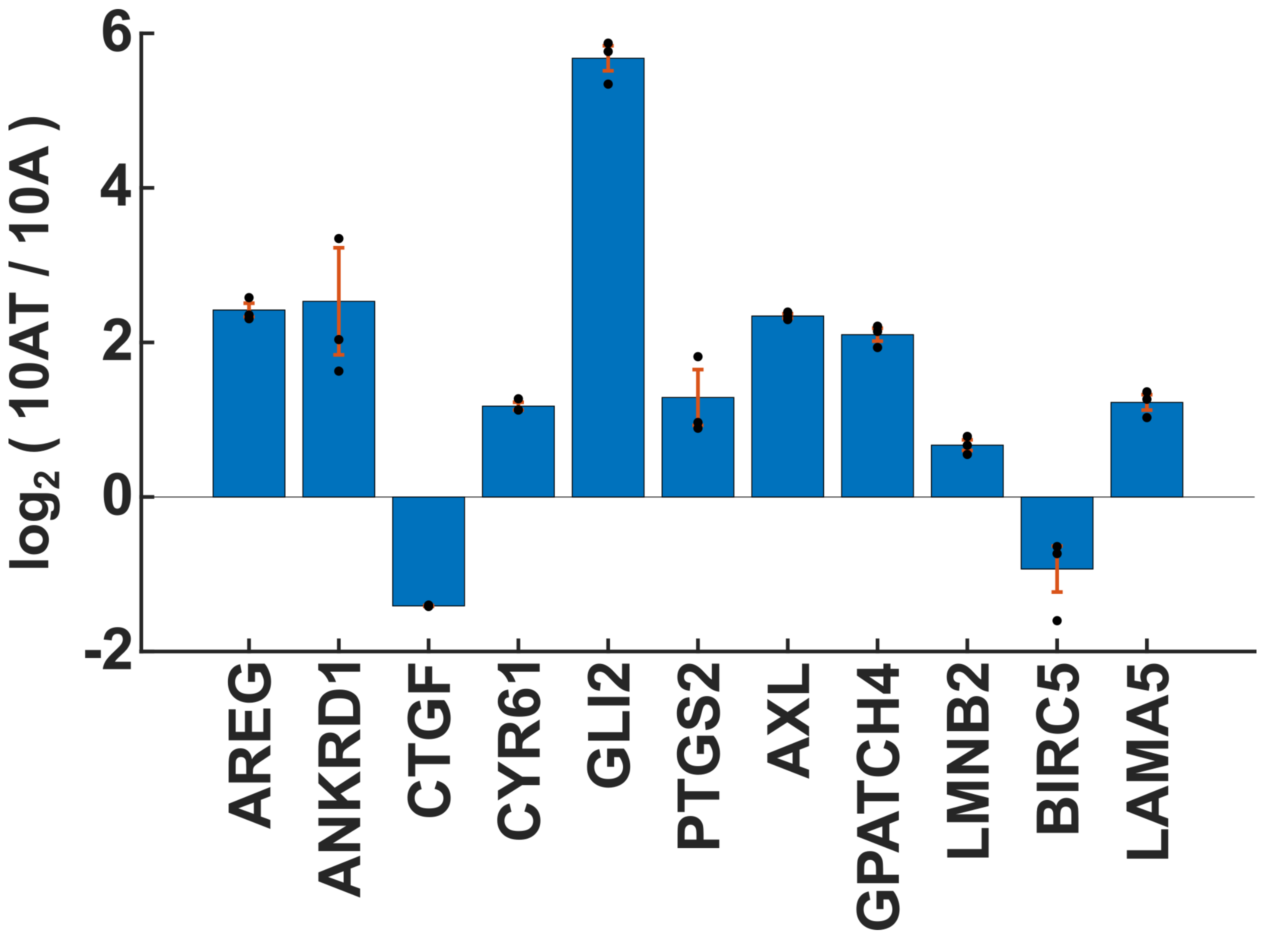

Supplementary Fig. 6: Transformed mammary epithelial cells exhibit higher expression of YAP target gene. Gene expression in Ras-transformed MCF10A (10AT) relative to MCF10A (10A) for genes known to be regulated by YAP activity based on reported RNA-seq measurements (See Methods)**. N=3 replicates for MCF10A and N=5 replicates for Ras-transformed MCF10A. Error bars are SEM.**

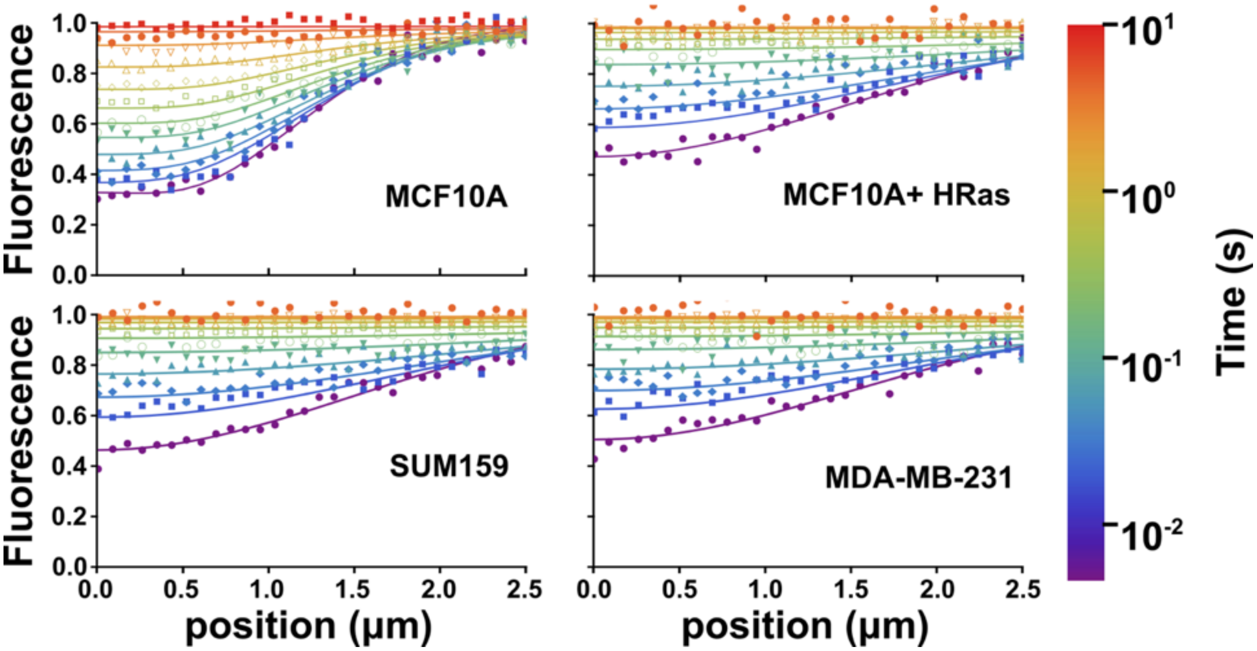

Supplementary Fig. 7: Line FRAP reveals binding kinetics of YAP in normal and transformed breast epithelial cell lines. A spatio-temporal FRAP model was used to fit high-resolution line FRAP experiments of YAP in MCF10A (N=30 cells), MCF10A+HRas (N=26 cells), SUM159 (N=30 cells), and MDA-MB-231 (N=30). Markers represent the measured mean fluorescence values as a function of time (color bar) and position from bleach center (x-axis). The model fit is shown in solid colored lines.

Supplementary Table 1. Guide sequences used for CRISPR knockin

YAP	TTAGAATTCAGTCTGCCTGA
TEAD	GGCTTGTAAAGGACTGAACA
AREG	AAGAAACTTCGACAAGAGAA
ANKRD1	TCTCGCATAGCTACATTCTG

Supplementary Table 2. Genomic PCR primers

Gene	fw primer (5'-3')	rev primer (5'-3')	wild-type amplicon (bp)	knock-in amplicon (bp)
YAP	ccttggaacactggaaggagatggaatgaacatag	cagcaatacattaacagcagcaatggacaaggaagag	305	1793
TEAD1	ggtggtaacaaacagggatacacaagaaactc	cctcggggccagggcaccaccctgtgtggtgagg	235	2181
*AREG***	tggatttatatacagtcaacataaataccccct	atactcaaagaataatactttaacatagccattgt	455	2336
ANKRD1	acagagaatgtgaatttacagatgtcacccacctg	ctagattttatggctagtgtctcttctcttgggcc	261	2154

****** Genomic PCR validation of the MS2 transcriptional cassette knockin for *AREG*, produced a slightly smaller amplicon than expected (Supplementary Figure 4b, lane 5). This can be attributed to the very high A/T content at the 5' and 3' regions surrounding the knockin site and the highly repetitive nature of the MS2 cassette. PCR of the homologous recombination vector using the same primer set produced an amplicon identical in size to the genomic PCR, even though restriction digestion and sequencing revealed correct junctions and MS2 cassette size. Moreover, smRNA FISH of MCF10A$^{\textit{AREG}\text{-MS2-KI}}$ with labeled oligonucleotides designed against native RNA sequence showed perfect overlap with nascent transcription site detected using MCP-mNeon (Figure 5a).

Supplementary Table 3. RT-qPCR primer list

CTGF (FW)	TTAAGAAGGGCAAAAAGTGC
CTGF (REV)	CATACTCCACAGAATTTAGCTC
GAPDH (FW)	ACAGTTGCCATGTAGACC
GAPDH (REV)	TTGAGCACAGGGTACTTTA
ANKRD1 (FW)	TGAGTATAAACGGACAGCTC
ANKRD1 (REV)	TATCACGGAATTCGATCTGG
AREG (FW)	AAAGAAAGAAAAAGGGAGGC
AREG (REV)	CATTTGCATGTTACTGCTTC
CYR61 (FW)	TTGATTGCAGTTGGAAAAGG
CYR61 (REV)	GCCTTGTAAAGGGTTGTATAG
IGFBP3 (FW)	AGAGCACAGATACCCAGAACT
IGFBP3 (REV)	GGTGATTCAGTGTGTCTTCCATT
TGFB2 (FW)	CAGCACACTCGATATGGACCA
TGFB2 (REV)	CCTCGGGCTCAGGATAGTCT
FOXF2 (FW)	CCCAGCATGTCTTCCTACTCG
FOXF2 (REV)	CGAAATCTTTCCTGTCGCACAC
RASSF2 (FW)	AAGAAGACGAGTTCATTGTGGAG
RASSF2 (REV)	GAATGCGTTCGTTGTCATCC
HPRT1 (FW)	TGACACTGGCAAAACAATGCA
HPRT1 (REV)	GGTCCTTTTCACCAGCAAGCT
PGK1 (FW)	GAACAAGGTTAAAGCCGAGCC
PGK1 (REV)	GTGGCAGATTGACTCCTACCA

Supplementary Table 4: Line-FRAP model fitting.

cell line	Pure diffusion		reaction-diffusion				
	D ($\mu m^2/s$)	SSD	D_{eff} ($\mu m^2/s$)	k_{on} (1/s)	k_{off} (1/s)	% Bound	SSD
MCF10A	**2.05±0.18**	8.82±0.65	9.8±2.4	1.19±0.41	1.06±0.19	52±5	7.97±0.59
MCF10A+HRas	23.5±4.1	6.51±0.74	**38.3±10.2**	0.26±0.26	1.12±0.52	16±7	6.23±0.68
SUM159	26.8±5.1	2.57±0.40	**40.8±10.9**	0.66±1.03	3.34±5.1	15±6	2.46±0.40
MDA-MB-231	26.5±7.7	3.56±0.44	**45.5±10.6**	0.52±0.47	1.99±1.2	19±7	3.41±0.42

Fitted model parameter values for MCF10A, MCF10A + HRas, SUM159, MDA-MB-231 for either a pure-diffusion or reaction-diffusion model. Unrealistic values are presented in bold. SSD is the sum of squared residuals between experiment data and model fit.
