# Supplementary material for: Concerted localization-resets precede YAP-dependent transcription
Source: Nat Commun. 2020 Sep 11;11:4581. doi: 10.1038/s41467-020-18368-x (PMC7486942; doi:10.1038/s41467-020-18368-x)
Supplement: Supplementary file 3 — Description of Additional Supplementary Files [file 41467_2020_18368_MOESM3_ESM.pdf]

Description of Additional Supplementary Files

File Name: Supplementary Movie 1
Description: 1.5 µM Latrunculin B treatment of MCF10A$^{YAP-GFP-KI}$ cells.

File Name: Supplementary Movie 2
Description: YAP localization vs. density in MCF10A$^{YAP-GFP-KI}$ and HRas transformed MCF10A$^{YAP-GFP-KI}$

File Name: Supplementary Movie 3
Description: Highlighted MCF10A$^{YAP-GFP-KI}$ cell with rapid changes of YAP localization.

File Name: Supplementary Movie 4
Description: YAP N/C localization in MCF10A$^{YAP-GFP-KI}$ cells, MCF10AT$^{YAP-GFP-KI}$ cells and MCF10A$^{YAP-GFP-KI}$ cells treated with the SRC inhibitor PP1.

File Name: Supplementary Movie 5
Description: Serum-starved MCF10A$^{YAP-GFP-KI}$ cells stimulated with serum-containing media.

File Name: Supplementary Movie 6
Description: H1$^{YAP-GFP-KI}$ hESC cells with a highlighted cell showing fluctuations in YAP localization.

File Name: Supplementary Movie 7
Description: YAP localization dynamics at the wound edge.

File Name: Supplementary Movie 8
Description: Thapsigargin-induced YAP localization in MCF10A control, Go6976 pre-treatment, and Δ50LaminA overexpression. Thapsigargin added before frame 1.

File Name: Supplementary Movie 9
Description: Thapsigargin induced nuclear-deformation comparison between control and Δ50LaminA expressing cells.

File Name: Supplementary Movie 10
Description: Tracking transcription kinetics of serum-starved MCF10A$^{AREG-MS2-KI}$ cells before and after stimulation with serum-containing media.

File Name: Supplementary Movie 11
Description: Accumulation of cytoplasmic ANKRD1 mRNAs in serum-starved MCF10A$^{Ankrd1-MS2-KI}$ cells stimulated with serum-containing media.

File Name: Supplementary Movie 12
Description: Tracking transcription kinetics before and after Thapsigargin treatment in MCF10A$^{ANKRD1-MS2-KI}$ and MCF10A$^{AREG-MS2-KI}$ cells.

File Name: Supplementary Movie 13
Description: Transcription kinetics before and after mitosis in MCF10A$^{ANKRD1-MS2-KI}$ and MCF10A$^{AREG-MS2-KI}$ cells. The timestamp is with respect to cytokinesis.
