# Supplementary material for: Concerted localization-resets precede YAP-dependent transcription
Source: Nat Commun. 2020 Sep 11;11:4581. doi: 10.1038/s41467-020-18368-x (PMC7486942; doi:10.1038/s41467-020-18368-x)
Supplement: Supplementary file 17 — Reporting Summary [file 41467_2020_18368_MOESM17_ESM.pdf]

nature research

Corresponding author(s): Rajarshi P Ghosh
Jan T Liphardt

Last updated by author(s): Jul 7, 2020

Reporting Summary

Nature Research wishes to improve the reproducibility of the work that we publish. This form provides structure for consistency and transparency in reporting. For further information on Nature Research policies, see our Editorial Policies and the Editorial Policy Checklist.

Statistics

For all statistical analyses, confirm that the following items are present in the figure legend, table legend, main text, or Methods section.

n/a	Confirmed	
☐	☒	The exact sample size (n) for each experimental group/condition, given as a discrete number and unit of measurement
☐	☒	A statement on whether measurements were taken from distinct samples or whether the same sample was measured repeatedly
☐	☒	The statistical test(s) used AND whether they are one- or two-sided<br>*Only common tests should be described solely by name; describe more complex techniques in the Methods section.*
☒	☐	A description of all covariates tested
☒	☐	A description of any assumptions or corrections, such as tests of normality and adjustment for multiple comparisons
☐	☒	A full description of the statistical parameters including central tendency (e.g. means) or other basic estimates (e.g. regression coefficient) AND variation (e.g. standard deviation) or associated estimates of uncertainty (e.g. confidence intervals)
☐	☒	For null hypothesis testing, the test statistic (e.g. F, t, r) with confidence intervals, effect sizes, degrees of freedom and P value noted<br>*Give P values as exact values whenever suitable.*
☒	☐	For Bayesian analysis, information on the choice of priors and Markov chain Monte Carlo settings
☒	☐	For hierarchical and complex designs, identification of the appropriate level for tests and full reporting of outcomes
☒	☐	Estimates of effect sizes (e.g. Cohen's d, Pearson's r), indicating how they were calculated

Our web collection on statistics for biologists contains articles on many of the points above.

Software and code

Policy information about availability of computer code

Data collection	Images were collected using either a Zeiss LSM700 or Olympus FV10i system. The data collected on Zeiss LSM700 used the Zen software, and data collected on on the Olympus FV10i used the Fluoview software.
Data analysis	Custom image analysis was performed using software developed in-house. See: https://github.com/jmfrank/track_analyzer<br>RNA-seq data was analyzed using custom software developed in house. For ImageJ, MacOSX compatible version (ImageJ bundled with Java 1.8.0_172) was used. The version numbers for cutadapt is 1.18 and HISAT is 2.1.

For manuscripts utilizing custom algorithms or software that are central to the research but not yet described in published literature, software must be made available to editors and reviewers. We strongly encourage code deposition in a community repository (e.g. GitHub). See the Nature Research guidelines for submitting code & software for further information.

Data

Policy information about availability of data

All manuscripts must include a data availability statement. This statement should provide the following information, where applicable:

- Accession codes, unique identifiers, or web links for publicly available datasets
- A list of figures that have associated raw data
- A description of any restrictions on data availability

All oligonucleotide and primer sequences used CRISPR knockin, Genomic PCR and RT-qPCR can be found in supplementary information. Image data files can be made available upon reasonable request.
Source data files for Figures 1 c, f, g; 2b, c, g; 3 a, c, d, e, h; 4 b, c, d, f; 5 d, e, f, g, l; 6 a-e, i-m; 7 d, e; 8 a, c and Supplementary Figures 2 b, c; 3 a-c, e, f, h; 5; 6; 7 have been provided.

Field-specific reporting

Please select the one below that is the best fit for your research. If you are not sure, read the appropriate sections before making your selection.

- [x] Life sciences
- [ ] Behavioural & social sciences
- [ ] Ecological, evolutionary & environmental sciences

For a reference copy of the document with all sections, see nature.com/documents/nr-reporting-summary-flat.pdf

Life sciences study design

All studies must disclose on these points even when the disclosure is negative.

Sample size	A priori sample size calculation was not performed. Sample size specific to experiments were based on existing standards used in literature. In general we severely oversample for each experiment. Spatiotemporal FRAP and inverse FLIP experiments were performed for each condition on ~40 cells from two different samples. Where relevant, all curves/numbers/plots are given with standard deviations or standard errors of the mean (see main text and figures). Fluctuation and sequestration experiments were performed on 100s of cells per condition from multiple samples over multiple days. All live-cell transcription experiments were performed on three independent samples.
Data exclusions	We curated imaging datasets by manually flagging cells that appeared unhealthy or abnormal. These flagged cells were excluded from downstream analysis. For MCF10A, 10AT, SUM159 and MDA MB231 healthy morphology is well documented. We excluded cells that did not conform to the established health morphology and in general discarded dishes that had cells of abnormal morphology.
Replication	All findings were replicated by independent experiments at minimum two times. All attempts at replication were successful.
Randomization	There was no bias in selecting cells for data acquisition.
Blinding	We decided that blinding was not relevant to the study. This is not a clinically based study and does not involve experiments that require blinding. The dynamic nature of our experiments required imaging one sample at a time, and we found the effects were so distinct, we did not need require blinding to prevent experimenter bias.

Reporting for specific materials, systems and methods

We require information from authors about some types of materials, experimental systems and methods used in many studies. Here, indicate whether each material, system or method listed is relevant to your study. If you are not sure if a list item applies to your research, read the appropriate section before selecting a response.

Materials & experimental systems

n/a	Involved in the study	
[ ]	[x]	Antibodies
[ ]	[x]	Eukaryotic cell lines
[x]	[ ]	Palaeontology and archaeology
[x]	[ ]	Animals and other organisms
[x]	[ ]	Human research participants
[x]	[ ]	Clinical data
[x]	[ ]	Dual use research of concern

Methods

n/a	Involved in the study	
[x]	[ ]	ChIP-seq
[x]	[ ]	Flow cytometry
[x]	[ ]	MRI-based neuroimaging

Antibodies

Antibodies used	anti-YAP Antibody (63.7) - Santa Cruz biotechnology. The catalog number for the YAP antibody used is sc-101199.<br>The Catalog # for Mouse IgG2a is A-21137<br>The details regarding dilutions have been provided in the Methods section.
Validation	anti-YAP Antibody (63.7) - (Santa Cruz biotechnology) is a highly cited antibody for staining YAP localization. In our work, we validated this antibody for localization in Figure 1c-d with our YAP-eGFP knockin.<br>Further details of YAP antibody validation can be found at https://www.scbt.com/p/yap-antibody-63-7<br>under product citation and review categories.<br>Product details can be further found at https://datasheets.scbt.com/sc-101199.pdf

Eukaryotic cell lines

Policy information about cell lines

Cell line source(s)	MCF10A and MCF10A+HRas were kind gifts from Professor Valerie M. Weaver. MDA-MB-231 was obtained from ATCC. SUM159 was obtained from Asterand (now bioivt). H1 hESC was obtained from WiCell (hPSCReg ID: WAe001-A).

	The original MCF10A cell line was obtained from ATCC (ATCC® CRL-10317™). MCF10AT was originally obtained from Karmanos Cancer Center and was also produced in-house through H-ras transformation of MCF10A cell line.
Authentication	None of these cell lines have been authenticated in our lab. However, it should be noted that cells were obtained from reliable well established sources and cell phenotypes were identical to published results.
Mycoplasma contamination	Cell lines used were not tested for mycoplasma contamination.
Commonly misidentified lines<br>(See ICLAC register)	No commonly misidentified cell lines were used.
