# Supplementary material for: Concerted localization-resets precede YAP-dependent transcription
Source: Nat Commun. 2020 Sep 11;11:4581. doi: 10.1038/s41467-020-18368-x (PMC7486942; doi:10.1038/s41467-020-18368-x)
Supplement: Supplementary file 18 — Source data [file 41467_2020_18368_MOESM18_ESM.zip › data/ReadMe.rtf]

First add the ‘code’ directory to your MATLAB path. Make sure to install the class “@track_analyzer’ properly. Change the first 3rd row of the csv file ‘image_experiments.csv’ to reflect the path of the ‘cell_lines’ folder. Many of the scripts used to generate the plots are provided in ‘scripts’. These will re-generate the scripts seen in the paper. 
